# The added value of ordinal analysis in clinical trials: an example in traumatic brain injury

**DOI:** 10.1186/cc10240

**Published:** 2011-05-17

**Authors:** Bob Roozenbeek, Hester F Lingsma, Pablo Perel, Phil Edwards, Ian Roberts, Gordon D Murray, Andrew IR Maas, Ewout W Steyerberg

**Affiliations:** 1 Department of Neurosurgery, Antwerp University Hospital, Wilrijkstraat 10, 2650 Edegem, Belgium; 2 Department of Public Health, Erasmus MC, P.O. Box 2040, 3000 CA Rotterdam, The Netherlands; 3 Epidemiology and Population Health Department, London School of Hygiene & Tropical Medicine, Keppel Street, London, WC1E 7HT, UK; 4 Centre for Population Health Sciences, University of Edinburgh, Teviot Place, Edinburgh, EH8 9AG, UK

## Abstract

**Introduction:**

In clinical trials, ordinal outcome measures are often dichotomized into two categories. In traumatic brain injury (TBI) the 5-point Glasgow outcome scale (GOS) is collapsed into unfavourable versus favourable outcome. Simulation studies have shown that exploiting the ordinal nature of the GOS increases chances of detecting treatment effects. The objective of this study is to quantify the benefits of ordinal analysis in the real-life situation of a large TBI trial.

**Methods:**

We used data from the CRASH trial that investigated the efficacy of corticosteroids in TBI patients (*n *= 9,554). We applied two techniques for ordinal analysis: proportional odds analysis and the sliding dichotomy approach, where the GOS is dichotomized at different cut-offs according to baseline prognostic risk. These approaches were compared to dichotomous analysis. The information density in each analysis was indicated by a Wald statistic. All analyses were adjusted for baseline characteristics.

**Results:**

Dichotomous analysis of the six-month GOS showed a non-significant treatment effect (OR = 1.09, 95% CI 0.98 to 1.21, *P *= 0.096). Ordinal analysis with proportional odds regression or sliding dichotomy showed highly statistically significant treatment effects (OR 1.15, 95% CI 1.06 to 1.25, *P *= 0.0007 and 1.19, 95% CI 1.08 to 1.30, *P *= 0.0002), with 2.05-fold and 2.56-fold higher information density compared to the dichotomous approach respectively.

**Conclusions:**

Analysis of the CRASH trial data confirmed that ordinal analysis of outcome substantially increases statistical power. We expect these results to hold for other fields of critical care medicine that use ordinal outcome measures and recommend that future trials adopt ordinal analyses. This will permit detection of smaller treatment effects.

## Introduction

Traumatic brain injury (TBI) is a major health and socio-economic problem throughout the world. Basic research has elucidated many of the pathophysiological mechanisms underpinning secondary damage and many neuroprotective agents have been developed to counteract these mechanisms. Since the 1980s, at least 33 randomized controlled phase III trials have been performed to investigate the effectiveness of new therapeutic interventions in TBI, but none has convincingly demonstrated benefit in the overall population [1]. Heterogeneity of the population and limitations of the conventional statistical analysis of TBI trials contribute to this lack of success [2,3]. We recently published a set of recommendations for improving the design and analysis of future TBI trials [4]. These recommendations were mainly derived from simulation studies and include the use of relatively broad enrolment criteria, covariate adjustment and ordinal rather than dichotomous outcome analysis.

In most phase III TBI trials, the 5-point Glasgow Outcome Scale is used as the primary outcome measure, usually measured at six months after injury, and dichotomized as unfavourable (Dead, Vegetative or Severe Disability) versus favourable outcome (Moderate Disability or Good Recovery) (Table 1). Similar approaches are often used in the analysis of trials conducted for other indications. For example, in stroke the modified Rankin scale, which is also an ordinal scale, consisting of six categories, is commonly collapsed into a binary scale. This dichotomous outcome is then analysed with a chi-squared test or with binary logistic regression. Simulation studies have demonstrated that ordinal outcome analysis in TBI trials can increase statistical power [5]. These results have not yet been validated in empirical data. The aim of this study is to investigate whether the benefits of an ordinal analysis would be upheld on analysis of the largest trial in TBI ever, which did demonstrate a true (but negative) treatment effect.

**Table 1:** The Glasgow Outcome Scale and its traditional dichotomy in favourable versus unfavourable outcome

Dead	
Vegetative State	Unfavourable
Severe Disability	
Moderate Disability	Favourable
Good Recovery	

## Materials and methods

### Data

We used the individual patient data of the MRC CRASH trial into which 10,008 patients were enrolled.

The CRASH trial (Corticosteroid Randomisation After Significant Head Injury) was an international, randomised, placebo-controlled trial designed to investigate the effect of early administration of methylprednisolone on the risk of death and disability after head injury. Full results have been reported [6,7]. Enrolment was stopped in May 2004, following demonstration of a higher 14-day mortality rate in the active treatment arm (21.1% versus 17.9% deaths; *P *= 0.0001). Outcome at six months was available for 9,554 patients. The current study was exempt from institutional review board approval.

### Conventional dichotomous outcome analysis

We first estimated the effect of the treatment on the six-month GOS, dichotomized as unfavourable versus favourable, with binary logistic regression. The treatment effect was adjusted for four baseline covariates: age, Glasgow Coma Scale (GCS), pupillary reactivity and presence of major extracranial injury. Age was handled as a continuous variable and GCS as a categorical variable (range 3 to 15). Pupillary reactivity was grouped into three categories: both pupils reactive, one reactive and none reactive to light. The presence of major extracranial injury was included as a binary variable, having a positive value when patients had an extracranial injury that required hospital admission on its own.

Subsequently, we used two approaches exploiting the ordinal nature of the GOS: a proportional odds logistic regression model and the sliding dichotomy approach.

### Proportional odds logistic regression

A proportional odds logistic regression model was fitted with the GOS collapsed to a 4-point ordinal scale (Severe Disability and Vegetative State were taken together) as the outcome variable. The proportional odds model has the same structure as the binary logistic regression model, but uses an ordinal outcome variable with more than two possible categories. It estimates a common odds ratio over all possible cut-offs of the outcome scale. The common odds ratio is formally valid if the odds ratios for each cut-off are the same (the proportional odds assumption). We can, however, interpret the common odds ratio as a summary measure of treatment effect, even if the odds ratios differ by cut-off [8]. The common odds ratio can also be interpreted as the average shift over the total ordinal outcome scale caused by the treatment under study [5,9,10].

### Sliding dichotomy

The sliding dichotomy approach dichotomizes the GOS into a binary measure, but the point of dichotomy is tailored to each individual patient's baseline prognosis [11]. For example, for a patient with an excellent prognosis only good recovery may be considered as a favourable outcome, whereas for a patient with a very poor prognosis, survival may be regarded as a favourable outcome. First, the baseline prognostic risk of each patient was estimated by calculating the probability of unfavourable outcome with a prediction model including the following variables: age, GCS, pupillary reactivity, and presence of major extracranial injury [12]. Subsequently, patients were divided into three prognostic bands of equal size, that is, for the best, intermediate and worst prognosis. For each band a separate cut-off on the GOS was defined and a new outcome variable was generated. For example, in the best prognosis band we only considered Good Recovery as a favourable outcome. The effect of treatment on this newly constructed dichotomous outcome was then estimated with binary logistic regression, with stratification by prognostic band and adjustment for the four covariates mentioned above. The pooled sliding dichotomy odds ratio can be interpreted as the effect of treatment on outcomes being worse than expected [11].

### Comparison of the different approaches

We calculated Wald statistics, based on the coefficients of the treatment effect and the corresponding standard error for each analysis. The ratio of the Wald statistics can be interpreted as the gain in information density and is, therefore, a suitable measure for the efficiency of the different approaches.

We adjusted the treatment effect for four baseline covariates in all analyses (age, GCS, pupillary reactivity, major extracranial injury) [12,13]. Missing data occurred for 509 patients on pupillary reactivity and 196 on the presence of extracranial injury. These missing covariates were imputed with a multiple imputation model. Statistical analyses were performed in R Statistical Software version 2.7.2 using the *Design *library (R Foundation for Statistical Computation, Vienna, Austria).

## Results

**Figure 1:**
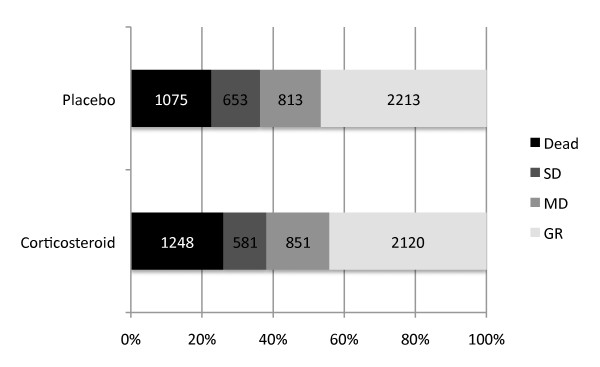
**Distribution of the Glasgow Outcome Score at six months after injury**. Data from the CRASH trial (*n *= 9,554). SD, severe disability (including vegetative state); MD, moderate disability; GR, good recovery

The CRASH trial included 10,008 patients. We excluded 454 patients with missing six-month GOS score, leaving 9,554 for the analyses. Median age was 33 years, and 81% of the patients were male (Table 2). At six months after injury, 2,323 (24%) patients had died and 3,557 (37%) had an unfavourable outcome (Figure 1). Dichotomous analysis of the six-month GOS showed a non-significant adjusted odds ratio (OR) of 1.09 (95% CI 0.98 to 1.21, *P *= 0.096).

**Table 2:** Baseline characteristics of patients enrolled in the CRASH trial with Glasgow Outcome Scale score available

	Corticosteroid (*n *= 4,800)	Placebo (*n *= 4,754)
**Age (median, IQR)**	33, 23 to 47	32, 23 to 48
**Gender**		
Male	3,892 (81.1%)	3,824 (80.4%)
**Glasgow Coma Scale**		
Severe (3 to 8)	1,925 (40.1%)	1,890 (39.8%)
Moderate (9 to 12)	1,477 (30.8%)	1,405 (29.6%)
Mild (13 to 14)	1,398 (29.1%)	1,459 (30.7%)
**Pupillary reactivity**		
Both reactive to light	3,860 (80.4%)	3,822 (80.4%)
One reactive to light	270 (5.6%)	294 (6.2%)
Both not reactive to light	412 (8.6%)	387 (8.1%)
Missing	258 (5.4%)	251 (5.3%)
**Major extracranial injury**		
Yes	1,106 (23.0%)	1,039 (21.9%)
No	3,600 (75.0%)	3,613 (76.0%)
Missing	94 (2.0%)	102 (2.1%)

IQR: interquartile range.

The use of different splits than the conventional favourable vs. unfavourable outcome resulted in rather different estimates of the treatment effect (Table 3). Further, the estimated treatment effect was non-significant when the conventional dichotomy was used, while it was significant when the split was taken at less than Good Recovery vs. Good Recovery (OR 1.12, 95% CI 1.01 to 1.23, *P *= 0.024) and death vs. survival (OR 1.27, 95% CI 1.13 to 1.43, *P *< 0.0001). Application of the proportional odds logistic regression model gave an estimated common odds ratio of 1.15 (95% CI 1.06 to 1.25) with a *P*-value of 0.0007.

**Table 3:** Analysis of the treatment effect according to different dichotomizations and proportional odds logistic regression

	Adjusted odds ratio^ (95% CI)	Wald statistic	*P*-value
Dichotomous odds ratios			
Less than good vs. good recovery	1.12 (1.01 to 1.23)	2.26	0.024
Unfavourable vs. favourable outcome	1.09 (0.98 to 1.21)	1.66	0.096
Death vs. survival	1.27 (1.13 to 1.43)	4.16	< 0.0001
Common odds ratio (proportional odds model)	1.15 (1.06 to 1.25)	3.41	0.0007

Analyses are based on the six-month Glasgow Outcome Scale. Data from the CRASH trial (*n *= 9,554).

An odds ratio > 1 indicates an adverse effect of corticosteroids.

^ Adjustment for age, GCS, pupillary reactivity and major extracranial injury

With the sliding dichotomy approach we divided the study population into three bands of equal numbers, based on the individual prognostic risk for unfavourable outcome of each patient (Table 4). For each prognostic band a different split for the dichotomization was used (better versus worse than expected). In the 'best prognosis' band the split was taken at Good Recovery versus worse than Good Recovery, in the 'intermediate prognosis' band at Moderate Disability or better versus Severe Disability or worse, and in the 'worst prognosis' band between death and survival. An unadjusted odds ratio was calculated for each prognostic band. These odds ratios varied between 1.06 (95% CI 0.91 to 1.23, *P *= 0.45) for the 'intermediate prognosis' band and 1.28 (95% CI 1.11 to 1.47, *P *= 0.0006) for the 'worst prognosis' band. Unadjusted and adjusted pooled odds ratios were similar (1.17, 95% CI 1.07 to 1.27, *P *= 0.0003 and 1.19, 95% CI 1.08 to 1.30, *P *= 0.0002).

**Table 4:** Analysis of the Glasgow Outcome Scale with the sliding dichotomy approach

		Dead	SD	MD	GR	Worse than expected	Better than expected	Odds ratio (95% CI)	Wald statistic	*P*-value
**Best prognosis**	Corticosteroid	67	86	274	1,162	427	1,162	1.22 (1.03 to 1.43)		0.017
	Placebo	59	84	228	1,227	371	1,227			
										
**Intermediate prognosis**	Corticosteroid	282	215	365	748	497	1,113	1.06 (0.91 to 1.23)		0.45
	Placebo	225	241	357	749	466	1106			
										
**Worst prognosis**	Corticosteroid	899	280	212	210	899	702	1.28 (1.11 to 1.47)		0.0006
	Placebo	791	328	228	237	791	793			
										
**Pooled odds ratio, unadjusted**	1.17 (1.07 to 1.27)								3.67	0.0003
**Pooled odds ratio, adjusted^**	1.19 (1.08 to 1.30)								3.69	0.0002

The prognosis bands were created with model containing the variables age, GCS, pupillary reactivity and major extracranial injury. Odds ratios were given by prognosis band, for the unadjusted treatment effect. Pooled odds ratios were given for the unadjusted and adjusted treatment effect. An odds ratio > 1 indicates an adverse effect of corticosteroids. Patients with better outcome than expected are underlined. Data from the CRASH trial (*n *= 9,554).

^ Adjustment for age; GCS, pupillary reactivity and major extracranial injury.

GCS, Glasgow Coma Scale; SD, Severe Disability (including Vegetative State); MD, Moderate Disability; GR, Good Recovery; OR, odds ratio.

The logistic regression analysis with dichotomized GOS resulted in a Wald statistic for the treatment effect of 1.66 (*P *= 0.096). Ordinal analysis with a proportional odds model gave a 2.05-fold higher Wald statistic (3.41, *P *= 0.0007). The sliding dichotomy approach resulted in an even larger Wald statistic of 3.69 (*P *= 0.0002), indicating a 2.56-fold increase in information density.

## Discussion

Analysis of the MRC CRASH trial data showed that ordinal analysis of the GOS resulted in substantially greater statistical power to detect a treatment effect with equal sample size. Whilst results obtained with the conventional analysis of the dichotomized GOS were non-significant, those obtained with ordinal analysis were highly significant. With ordinal analysis, a 2- to 2.5-fold gain in information density was demonstrated, compared to the dichotomized analysis. Simulation studies had already suggested the potential for ordinal analysis to increase statistical power in TBI trials, but our current study has proven the value of this approach in the empirical data of a large trial with a true treatment effect.

Earlier research has demonstrated that adjustment for strong predictors of outcome (covariate adjustment) may result in a substantial increase in statistical power and trial efficiency [13-15]. In the IMPACT database, we found that the required sample size for a RCT could potentially be reduced by around 25% when covariate adjustment would be applied with seven strong predictors [13]. We, therefore, incorporated covariate adjustment in all analyses in the present study.

Why is the use of ordinal outcome analysis beneficial? The common practice of collapsing an ordinal outcome measure to a binary scale results in a loss of information [16]. Moreover, dichotomization gives priority to one particular transition in the outcome scale: in the case of the GOS this is the change from severe disability to moderate disability. Patients with a relatively extreme prognosis have little potential to contribute to the detection of a treatment effect on an ordinal functional outcome scale, when this scale is dichotomized for the analysis [17]. A patient with a very good prognosis will almost inevitably have a favourable outcome, even without the benefits of a new effective therapy. In contrast, for patients with a very poor prognosis it is extremely unlikely to have a favourable outcome at six months, even with a very beneficial new treatment. This does not mean that these patients may not benefit from the treatment, but simply that the fixed split for dichotomising the outcome measure is not appropriate for these situations. When the outcome is analysed in an ordinal way, all patients can contribute to the detection of a treatment effect.

The idea of exploiting the ordinal nature of ordered outcome scales is far from a new concept in the statistical community [18]. Nevertheless, this approach has not been applied to the analysis of clinical trials on a regular basis. The sliding dichotomy approach was recently applied for the primary efficacy in a number of trials: the PAIS trial in stroke [19], the STICH trial in spontaneous intracerebral hemorrhage [20], and the Pharmos trial in TBI [21]. The proportional odds model was used in several neurological trials, for example, in the GAIN International trial [22] and the SAINT I trial [23].

Inherent to the proportional odds model is the proportional odds assumption, that is, that the treatment effect is constant across all cut-offs of the outcome scale. This assumption may partly be violated in empirical data. We, therefore, recommend reporting the odds ratios per cut-off if a common odds ratio is reported as the summary measure of the treatment effect. Indeed, we found that the odds ratios were not identical across all cut-offs for the GOS (Table 2). Also, some variation was seen in the odds ratios across prognostic bands for the sliding dichotomy (Table 3). The proportional odds assumption was formally tested with the 'PROC LOGISTIC' test from the SAS software package (SAS Institute Inc., Cary, NC, USA) and was found to be violated. This was confirmed by a graphical test in R software (the 'residuals' function from the *Design *library) to test for parallelism. In a previous study we simulated a non-proportional treatment effect, that is, a treatment that only affected mortality and did not cause a shift for the other categories of the GOS. We found to our surprise that the statistical power of ordinal analyses (proportional odds or sliding dichotomy) remained higher than a dichotomous analysis at the 'correct' cut-off (mortality vs. survival) [11]. This robust gain in statistical power is a clear advantage of ordinal analysis, even if one were to object to interpretation of a summary odds ratio when underlying assumptions are violated [8].

The choice between the two ordinal approaches involves primarily a value judgement. The sliding dichotomy approach and its explanation (the effect of treatment on outcomes being worse than expected) may be particularly appealing for clinicians, but it requires a (validated) prognostic model to identify each patient's baseline prognostic risk. The proportional odds method does not necessarily require such a model, but may not have a proper interpretation if effect estimates vary substantially by cut-off (a violation of the proportional odds assumption). A pragmatic approach is to focus more on the underlying concept of 'shift analysis', instead of emphasizing the formal assumptions of this model.

Both approaches to ordinal outcome analysis that were investigated in the present study resulted in substantial power increase. Therefore, we strongly recommend incorporating ordinal methods in the analysis of future trials when an ordered outcome measure is considered. We do not advocate that this power increase should motivate reduced sample sizes for future trials. Since most TBI trials that were published in the past decades have been underpowered [24], the power increase that results from ordinal analysis can thus be used to increase the chance of detecting smaller, but clinically relevant, treatment effects with the same sample size.

The use of ordinal outcome scales is not unique to TBI, but is common to many fields of clinical research. Equally common is the practice of dichotomising ordinal outcome measures. In the field of stroke research, the modified Rankin Scale and the Barthel Index are often used as primary efficacy endpoints - and are also dichotomized [25,26]. The Optimising Analysis of Stroke Trials (OAST) Collaboration has shown the benefit of ordinal analysis in the field of stroke [27]. Other examples of ordinal outcome scales can be found in cardiology (for example, NYHA Functional Classification for heart failure), vascular surgery (for example, Rutherford Classification for peripheral artery disease) and pain management (for example, Visual Analogue Scale). The widespread use of ordinal outcome measures and the persisting practice of collapsing these measures into a binary outcome indicate that our findings in this case study on TBI have much broader implications than for TBI alone. We consider our results directly relevant to clinical trials in other fields of medicine that use ordinal outcome measures, especially if outcomes occur over the full range of the scale.

## Conclusions

We conclude that the application of ordinal outcome analysis substantially increases the power of a clinical trial. We recommend that future randomized trials, which use an ordinal outcome measure as efficacy parameter, adopt ordinal outcome analysis in order to facilitate detection of smaller treatment effects.

## Key messages

• None of the phase III clinical trials for Traumatic Brain Injury (TBI) has shown an overall significant treatment effect. Inefficient analysis of trials may contribute this the failure.

• Dichotomous analysis of an ordinal outcome scale in clinical trials results in loss of information. Previous simulation studies suggested that ordinal outcome analysis could substantially improve statistical power of a clinical TBI trial.

• The present study gives a real-life example of the benefit two approaches to ordinal outcome analysis in a large TBI trial (the CRASH trial).

• Both approaches to ordinal analysis showed highly significant treatment effects, increased statistical power and a 2.1- to 2.6-fold increase in information density.

• We recommend that future trials adopt ordinal outcome analysis, in order to facilitate detection of smaller treatment effects.

## Abbreviations

CI: confidence interval; CRASH: Corticosteroid Randomisation After Significant Head Injury; GCS: Glasgow Coma Scale; GOS: Glasgow Outcome Scale; IMPACT: International Mission on Prognosis and Clinical trial design in TBI; MRC: Medical Research Council; NYHA: New York Heart Association; OAST: Optimizing Analysis of Stroke Trials; OR: odds ratio; PAIS: Paracetamol (Acetaminophen) Ischemic Stroke; RCT: randomized controlled trial; SAINT: Stroke-Acute Ischemic NXY Treatment; STICH: Surgical Trial in Intracerebral Haemorrhage; TBI: traumatic brain injury

## Competing interests

The authors declare that they have no competing interests.

## Authors' contributions

BR and HFL performed the analyses under supervision of EWS. BR wrote the first version of this manuscript. PP, PE and IR prepared and provided the CRASH trial data. EWS, GDM and AIRM developed the outline for the study. All authors provided critical comments on previous versions of this manuscript.
